# Supplementary material for: Seasonal Characteristics and Particle-size Distributions of Particulate Air Pollutants in Urumqi
Source: Int J Environ Res Public Health. 2019 Jan 31;16(3):396. doi: 10.3390/ijerph16030396 (PMC6388106; doi:10.3390/ijerph16030396)
Supplement: Supplementary file 1 [file ijerph-16-00396-s001.pdf]

Table S1 Categories of air based on air quality index (AQI) (Mintz, D. 2009).

Range of AQI	Air quality conditions/color code	Effect on health	Suggestive measures
0-50	Good/green	There are no health concerns, since the quality of air is good.	All kinds of people are allowed to take normal activities.
51-100	Moderate/yellow	Since the quality of air is moderate, there are no health concerns and it is considered to be safe for most people. There might be a weak effect on health of some highly sensitive people.	Such sensitive people should reduce outdoor activities.
101-150	Unhealthy for sensitive groups/orange	Aggravation of symptoms of susceptible people who may be influenced on the heart and respiratory system.	Children, the elderly, and patients with heart disease and respiratory disease should reduce long-time and high-strength outdoor exercises.
151-200	Unhealthy/red	Dramatic aggravation of symptoms of vulnerable groups who may be influenced on the heart and respiratory systems.	Children, the elderly, and patients with heart disease and respiratory disease should avoid long-time and high-strength outdoor exercises; common people should reduce outdoor exercises.
201-300	Very unhealthy/purple	Aggravation of symptoms of people with heart attack and pulmonary disease who may have their exercise tolerance degraded; common healthy people may suffer from certain symptoms.	Children, the elderly, and patients with heart disease and respiratory disease should stay indoors and avoid outdoor exercises; common people should reduce outdoor exercises.
>300	Hazardous/maroon	Healthy people have their exercise tolerance degraded, suffer obvious symptoms, and have some diseases breaking out in advance.	Children, the elderly, and patients should stay indoors and avoid physical consumption; common people avoid outdoor activities.
